# Inanspruchnahme zahnärztlicher Kontrolluntersuchungen in Deutschland – Prävalenzen und Trends auf Basis von GKV-Routinedaten

**DOI:** 10.1007/s00103-026-04253-3

**Published:** 2026-06-09

**Authors:** Laura Krause, Stefanie Seeling, Lukas Reitzle

**Affiliations:** Abteilung für Epidemiologie und Gesundheitsmonitoring, Robert Koch-Institut, Gerichtstr. 27, 13347 Berlin, Deutschland

**Keywords:** Zahnvorsorge, Kontrolluntersuchung, Gesetzliche Krankenversicherung, Routinedaten, Sekundärdatenanalyse, Dental care, Check-ups, Statutory health insurance, Routine data, Secondary data analysis

## Abstract

**Einleitung:**

Zahnärztliche Kontrolluntersuchungen tragen wesentlich zur Verringerung der oralen Krankheitslast bei. Dieser Beitrag untersucht erstmals für Deutschland die Inanspruchnahme zahnärztlicher Kontrolluntersuchungen durch Erwachsene ab 20 Jahren auf der Basis von Routinedaten.

**Methoden:**

Für die Analysen wurden die vertragszahnärztlichen Abrechnungsdaten gemäß BEMA (Einheitlicher Bewertungsmaßstab für zahnärztliche Leistungen) der Kassenzahnärztlichen Bundesvereinigung verwendet. Eine Inanspruchnahme zahnärztlicher Kontrolluntersuchung wurde angenommen, wenn mindestens eine Abrechnung der BEMA 01, 151, 152, 153, 154 oder 155 im Kalenderjahr dokumentiert war. Die Schätzung der Inanspruchnahme erfolgte bezogen auf alle gesetzlich Versicherten gemäß KM6-Statistik für die Jahre 2015 bis 2024 stratifiziert nach Geschlecht, Alter und Region in einem querschnittlichen Studiendesign.

**Ergebnisse:**

Die Quote der Inanspruchnahme zahnärztlicher Kontrolluntersuchungen verlief bis 2019 mit rund 64 % relativ konstant, mit einem Rückgang im ersten COVID-19-Pandemiejahr 2020 (62,1 %) und einem allmählichen Anstieg in den Folgejahren bis auf das Ausgangsniveau im Jahr 2024. Im Vergleich zur Gesamtquote wiesen Männer, Personen im jungen und frühen mittleren Erwachsenenalter, Hochaltrige sowie Personen in Westdeutschland eine geringere Inanspruchnahmequote auf. Im Zeitverlauf haben sich die Ost-West-Unterschiede in der Inanspruchnahmequote verringert.

**Diskussion:**

Mehr als jede dritte Person hat in 2024 keine zahnärztliche Kontrolluntersuchung in Anspruch genommen. Damit unterstreichen die Ergebnisse die Notwendigkeit präventiver Maßnahmen. Für Deutschland liegen bislang keine routinedatenbasierten Analysen zur Inanspruchnahme zahnärztlicher Kontrolluntersuchungen vor. Dieser Artikel schließt somit eine Forschungslücke.

**Zusatzmaterial online:**

Zusätzliche Informationen sind in der Online-Version dieses Artikels (10.1007/s00103-026-04253-3) enthalten.

## Abstract (en)

**Introduction:**

Dental check-ups contribute significantly to reducing the burden of oral disease. This article examines for the first time in Germany the utilisation of dental check-ups by adults aged 20 years and older based on routine data.

**Methods:**

The analyses were based on the outpatient dental claims data in accordance with BEMA (Uniform Assessment Scale for Dental Services) of the National Association of Statutory Health Insurance Dentists. A dental check-up was assumed to have been performed if at least one billing of BEMA 01, 151, 152, 153, 154 or 155 was documented in a calendar year. The utilisation was estimated as a proportion of all persons with statutory health insurance according to the KM6 statistics for the years 2015 to 2024, stratified by sex, age and region in a cross-sectional study design.

**Results:**

The rate of utilisation of dental check-ups remained relatively constant at around 64% until 2019, with a decline in the first year of the COVID-19 pandemic in 2020 (62.1%) and a gradual increase in the subsequent years to the initial level in 2024. Compared to the overall rate, men, young and early middle-aged adults, the very old and people in western Germany had a lower utilisation rate. Over time, the east–west differences in the utilisation rate in Germany have decreased.

**Discussion:**

More than one in three persons did not have a dental check-up in 2024. The results therefore highlight the need for preventive measures. For Germany, there had previously been no analyses of the utilisation of dental check-ups based on routine data. This article therefore fills a research gap.

## Einleitung

Zahnärztliche Kontrolluntersuchungen tragen wesentlich zur Verringerung der oralen Krankheitslast in der Bevölkerung bei [1, 2]. Im Rahmen dieser Vorsorgeuntersuchung können Personen mit einem hohen Risiko für orale Erkrankungen identifiziert und entsprechende Maßnahmen eingeleitet werden [1]. Eine regelmäßige Inanspruchnahme zahnärztlicher Kontrolluntersuchungen steht mit einer besseren Mundgesundheit und einer geringeren Rate an Karies, Parodontitis und Zahnverlust in Zusammenhang [2]. Dies ist von großer Bedeutung, da eine schlechte Mundgesundheit in Wechselwirkung mit nichtübertragbaren Krankheiten steht, wie beispielsweise Diabetes mellitus, Herz-Kreislauf-Erkrankungen und chronischen Atemwegserkrankungen [3, 4].

Gesetzlich Versicherte ab 18 Jahren haben 2‑mal im Jahr Anspruch auf eine zahnärztliche Kontrolluntersuchung [5]. Diese umfasst eine eingehende Untersuchung zur Feststellung von Zahn‑, Mund- und Kieferkrankheiten, eine Anleitung zur effektiven Mundhygiene, Hinweise zur Reduktion von Risikofaktoren, wie Tabakkonsum und zuckerhaltiger Ernährung, sowie bei Bedarf die Entfernung harter Zahnbeläge [5].

Vor diesem Hintergrund ist von Interesse, in welchem Umfang zahnärztliche Kontrolluntersuchungen von der erwachsenen Bevölkerung tatsächlich in Anspruch genommen werden. Für die Abrechnung der Kontrolluntersuchung mit der Gesetzlichen Krankenversicherung (GKV) durch Zahnärzt:innen sieht der Einheitliche Bewertungsmaßstab für zahnärztliche Leistungen (BEMA) die Nummer 01 vor (Untersuchung, ggf. Beratung, keine Mundhygieneanleitung; Tab. 1; [6]).

**Tab. 1:** Einheitlicher Bewertungsmaßstab für zahnärztliche Leistungen (*BEMA*): Gebührennummern zur Abrechnung zahnärztlicher Kontrolluntersuchungen bei Erwachsenen mit der Gesetzlichen Krankenversicherung (*GKV;* [6])

BEMA	Codierte Leistung	
01	Eingehende Untersuchung zur Feststellung von Zahn‑, Mund- und Kieferkrankheiten, einschließlich Beratung	
151	Besuch eines Versicherten, einschließlich Beratung und eingehender Unterweisung	
152	a)	Besuch je weiteren Versicherten in derselben häuslichen Gemeinschaft in unmittelbarem zeitlichen Zusammenhang mit einer Leistung nach Nummer 151 – einschließlich Beratung und eingehende Untersuchung
	b)	Besuch je weiteren Versicherten in derselben Einrichtung in unmittelbarem zeitlichen Zusammenhang mit einer Leistung nach Nummer 151 – einschließlich Beratung und eingehende Untersuchung
153	a)	Besuch eines Versicherten in einer Einrichtung zu vorher vereinbarten Zeiten und bei regelmäßiger Tätigkeit in der Einrichtung, einschließlich Beratung und eingehende Untersuchung, ohne Vorliegen eines Kooperationsvertrags nach § 119b Abs. 1 SGB V
	b)	Besuch je weiteren Versicherten in derselben Einrichtung in unmittelbarem zeitlichen Zusammenhang mit einer Leistung nach Nummer 153 a zu vorher vereinbarten Zeiten und bei regelmäßiger Tätigkeit in der Einrichtung, einschließlich Beratung
154	Besuch eines pflegebedürftigen Versicherten in einer stationären Pflegeeinrichtung (§ 71 Abs. 2 SGB XI) im Rahmen eines Kooperationsvertrags nach § 119b Abs. 1 SGB V, einschließlich Beratung und eingehende Untersuchung	
155	Besuch je weiteren pflegebedürftigen Versicherten in derselben stationären Pflegeeinrichtung (§ 71 Abs. 2 SGB XI) im Rahmen eines Kooperationsvertrags nach § 119b Abs. 1 SGB V, in unmittelbarem zeitlichen Zusammenhang mit einer Leistung nach Nr. 154	

*SGB* Sozialgesetzbuch, *Abs.* Absatz

Die Leistung nach BEMA 01 kann bei Erwachsenen je Kalenderhalbjahr einmal abgerechnet werden, frühestens nach Ablauf von 4 Monaten. Einmal im Jahr wird sie in einem Bonusheft dokumentiert, um im Bedarfsfall Anspruch auf einen höheren Festzuschuss zum Zahnersatz zu erhalten [7]. Bis September 2020 entsprach der einfache Festzuschuss rund 50 % der Gesamtkosten für die entsprechende Regelversorgung. Die Festzuschüsse erhöhten sich um 20 % des Ausgangswerts bei einem über 5 Jahre bzw. 30 % bei einem über 10 Jahre lückenlos geführten Bonusheft. Zum Oktober 2020 änderte der Gesetzgeber die Festzuschüsse sowie Bonusregelung und hob den Kassenanteil an den Gesamtkosten für die Regelversorgung auf 60 % an [8]. Dieser Anteil steigt auf 70 % bei einem über 5 Jahre bzw. 75 % bei einem über 10 Jahre lückenlos geführten Bonusheft. Für das erste Jahr der COVID-19-Pandemie 2020 sichert eine Ausnahmeregelung den Erhalt der Bonusleistung, wenn in diesem Jahr keine Kontrolle stattgefunden hat, darüber hinaus aber ein vollständig geführtes Bonusheft vorgelegt werden kann [8].

Für Haus- bzw. Heimbesuche bestehen gesonderte Regelungen. Finden zahnärztliche Kontrolluntersuchungen außerhalb der Praxis statt, z. B. in einer Pflegeeinrichtung oder im häuslichen Umfeld, haben Zahnärzt:innen die Möglichkeit, die BEMA-Positionen 151 bis 155 (Tab. 1) abzurechnen [6]. Im Gegensatz zur BEMA 01 werden diese Gebührennummern nicht im Bonusheft dokumentiert.

Ziel dieser Arbeit ist es, auf Basis der vertragszahnärztlichen Abrechnungsdaten der Kassenzahnärztlichen Bundesvereinigung (KZBV) für die Jahre 2015 bis 2024 den Anteil an Erwachsenen darzustellen, bei denen mindestens einmal im Jahr eine Leistung nach Nr. 01, 151, 152, 153, 154 oder 155 abgerechnet wurde. Dabei wird nach Geschlecht, Alter und Region (Ost/West) stratifiziert. Weiterhin werden Unterschiede nach Bundesland für das Jahr 2024 aufgezeigt. Soweit bekannt, liegt für Deutschland bisher keine Analyse zur Inanspruchnahme zahnärztlicher Kontrolluntersuchungen auf Basis von Routinedaten vor. Dieser Beitrag schließt somit eine Forschungslücke.

## Methoden

### Datengrundlagen und Studiendesign

Für die Analyse zur Inanspruchnahme zahnärztlicher Kontrolluntersuchungen wurden Daten der vertragszahnärztlichen Versorgung der KZBV der Jahre 2015 bis 2024 verwendet. Diese umfassen die Abrechnungsdaten aller gesetzlich Krankenversicherten in Deutschland gemäß BEMA. Neben den Angaben zu abgerechneten Leistungen sind Informationen zu Geschlecht, Alter und Wohnort der Versicherten dokumentiert. Zusätzlich wurde zur Bildung der Bezugspopulation die Statistik über Versicherte, gegliedert nach Status, Alter, Wohnort und Kassenart (Stichtag: 1. Juli des jeweiligen Jahres), auch KM6-Statistik genannt, herangezogen [9].

Die Schätzung der Inanspruchnahme zahnärztlicher Kontrolluntersuchungen erfolgte für die Jahre 2015 bis 2024 je Kalenderjahr in einem querschnittlichen Studiendesign. Eingeschlossen wurden alle Versicherten, die im jeweiligen Kalenderjahr 20 Jahre oder älter waren sowie gültige Werte für Alter, Geschlecht und Bundesland des Wohnorts aufwiesen. Die Altersgrenze ab 20 Jahren wurde gewählt, da die KM6-Statistik Personen zwischen 18 und 19 Jahren nicht einzeln ausweist. Eine Inanspruchnahme zahnärztlicher Kontrolluntersuchung wurde angenommen, wenn mindestens einmal eine Abrechnung der BEMA 01, 151, 152, 153, 154 oder 155 im jeweiligen Kalenderjahr dokumentiert war (Hauptanalyse). In einer Sensitivitätsanalyse wurde die Auswertung auf die Abrechnung der BEMA 01 beschränkt. In einer weiteren Sensitivitätsanalyse wurde von der KZBV zusätzlich zu den in die Hauptanalyse eingeschlossenen Gebührenziffern die BEMA Ä1 berücksichtigt. Diese beinhaltet die „Beratung eines Kranken, auch fernmündlich“ [10]. Da bei dieser Beratungsleistung keine eingehende Untersuchung stattfindet, wurde die Ä1 nicht in die Hauptanalyse eingeschlossen.

### Statistische Analysen

Von der KZBV wurde die Anzahl der Versicherten mit Inanspruchnahme zahnärztlicher Kontrolluntersuchungen stratifiziert nach Kalenderjahr, Geschlecht, Altersgruppe (junges Erwachsenenalter 20–34 Jahre, frühes mittleres Erwachsenenalter 35–44 Jahre, spätes mittleres Erwachsenenalter 45–64 Jahre, höheres Erwachsenenalter 65–74 Jahre, Hochaltrige ab 75 Jahre) und Bundesland des Wohnorts anonymisiert in aggregierter Form (ohne Personenbezug) bereitgestellt. Zur Berechnung des Anteils der Inanspruchnahme zahnärztlicher Kontrolluntersuchungen an allen gesetzlich Versicherten wurde die Anzahl der Versicherten mit Abrechnung der entsprechenden Gebührenposition ins Verhältnis zu allen GKV-Versicherten gemäß der KM6-Statistik [9] gesetzt. Die Inanspruchnahme wurde getrennt nach Geschlecht, Altersgruppe, Bundesland und Kalenderjahr berechnet. Die Analyse erfolgte gemäß Empfehlungen der Guten Epidemiologischen Praxis (GEP) und der Guten Praxis Sekundärdatenanalyse (GPS). Ein Auswertungsplan wurde vorab zur Analyse der Daten erstellt und mit dem Antrag auf Datennutzung bei der KZBV am 29.01.2025 eingereicht.

## Ergebnisse

### Inanspruchnahme zahnärztlicher Kontrolluntersuchungen

**Abb. 1:**
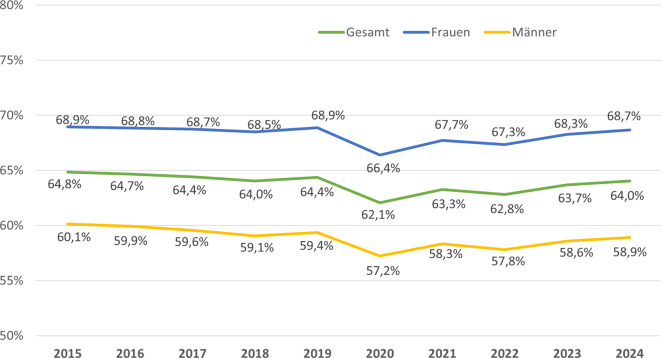
Zeitlicher Verlauf der Inanspruchnahme zahnärztlicher Kontrolluntersuchungen (mindestens eine Abrechnung der BEMA 01, 151, 152, 153, 154 oder 155 im Kalenderjahr) nach Geschlecht bei Erwachsenen ab 20 Jahren 2015 bis 2024; in Prozent (%). Quelle: vertragszahnärztliche Abrechnungsdaten der Kassenzahnärztlichen Bundesvereinigung (*KZBV*) und KM6-Statistik. (*BEMA* Einheitlicher Bewertungsmaßstab für zahnärztliche Leistungen)

Die Quote der Inanspruchnahme zahnärztlicher Kontrolluntersuchungen lag in den Jahren von 2015 bis 2019 konstant bei rund 64 % (Abb. 1). In 2020, dem ersten Jahr der COVID-19-Pandemie, zeigte sich ein Rückgang um etwa 2 Prozentpunkte. In den Folgejahren stieg die Inanspruchnahmequote sukzessive wieder an und erreichte im Jahr 2024 mit 64 % das präpandemische Niveau. Dieser Verlauf zeigte sich sowohl für Frauen als auch für Männer. Bei Männern wurde 2024 das präpandemische Niveau jedoch noch nicht vollständig erreicht. Die Quote der Inanspruchnahme zahnärztlicher Kontrolluntersuchungen lag im gesamten Untersuchungszeitraum bei Frauen deutlich höher als bei Männern. In 2024 betrug der Geschlechterunterschied rund 10 Prozentpunkte.

**Abb. 2:**
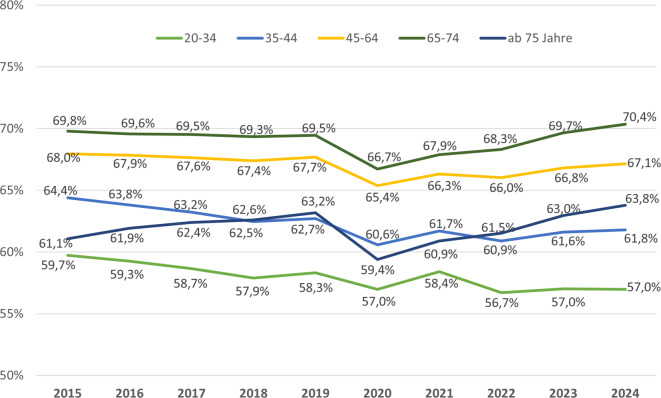
Zeitlicher Verlauf der Inanspruchnahme zahnärztlicher Kontrolluntersuchungen (mindestens eine Abrechnung der BEMA 01, 151, 152, 153, 154 oder 155 im Kalenderjahr) nach Alter bei Erwachsenen ab 20 Jahren 2015 bis 2024; in Prozent (%). Quelle: vertragszahnärztliche Abrechnungsdaten der Kassenzahnärztlichen Bundesvereinigung (*KZBV*) und KM6-Statistik. (*BEMA* Einheitlicher Bewertungsmaßstab für zahnärztliche Leistungen)

In den Jahren von 2015 bis 2019 lag die Quote der Inanspruchnahme zahnärztlicher Kontrolluntersuchungen bei Personen im späten mittleren (45–64 Jahre) und höheren Erwachsenenalter (65–74 Jahre) konstant bei knapp unter 70,0 % (Abb. 2). Bei Personen im jungen (20–34 Jahre) und frühen mittleren Erwachsenenalter (35–44 Jahre) nahm die Quote in diesem Zeitraum von knapp unter 60,0 % bzw. 65,0 % um rund 2 Prozentpunkte ab, während sie bei den Hochaltrigen (ab 75 Jahre), ausgehend von einem niedrigen Niveau (59,7 % im Jahr 2015), um etwas mehr als 3 Prozentpunkte anstieg. Der Rückgang in der Inanspruchnahmequote im ersten Jahr der COVID-19-Pandemie 2020 war bei allen Altersgruppen zu beobachten, am stärksten fiel dieser mit etwa 4 Prozentpunkten bei den Hochaltrigen (ab 75 Jahre) aus. Bei den 3 ältesten Altersgruppen stieg die Inanspruchnahmequote in den Folgejahren allmählich wieder an und erreichte das präpandemische Niveau oder lag teilweise leicht darüber. Bei den beiden jüngsten Altersgruppen stieg die Quote zunächst an, sank dann wieder ab und lag im Jahr 2024 unter dem präpandemischen Niveau. Im gesamten Untersuchungszeitraum wiesen junge Erwachsene (20–34 Jahre) die geringste Inanspruchnahmequote auf. Aber auch bei Personen im frühen mittleren Erwachsenenalter (35–44 Jahre) und bei Hochaltrigen (ab 75 Jahren) lag die Quote in fast allen Kalenderjahren unter der Gesamtquote von rund 64 % (Abb. 1 und 2).

**Abb. 3:**
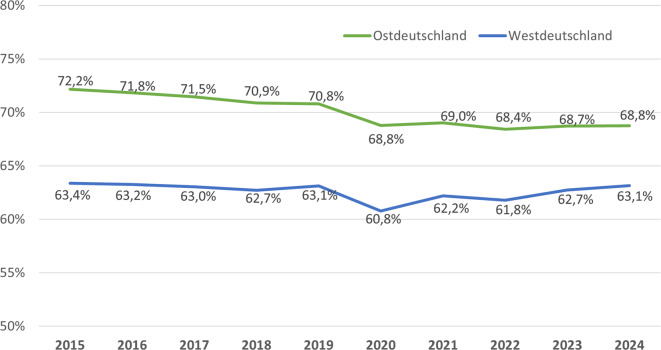
Zeitlicher Verlauf der Inanspruchnahme zahnärztlicher Kontrolluntersuchungen (mindestens eine Abrechnung der BEMA 01, 151, 152, 153, 154 oder 155 im Kalenderjahr) nach Region (Ost/West) bei Erwachsenen ab 20 Jahren 2015 bis 2024; in Prozent (%). Quelle: vertragszahnärztliche Abrechnungsdaten der Kassenzahnärztlichen Bundesvereinigung (*KZBV*) und KM6-Statistik. (*BEMA* Einheitlicher Bewertungsmaßstab für zahnärztliche Leistungen)

In Ostdeutschland war die Quote der Inanspruchnahme zahnärztlicher Kontrolluntersuchungen von 2015 bis 2019 rückläufig (2019: 70,8 %), während sie in Westdeutschland mit rund 63 % konstant verlief (Abb. 3). Im ersten Jahr der COVID-19-Pandemie (2020) sank die Inanspruchnahmequote in beiden Landesteilen um etwa 2 Prozentpunkte. In den Folgejahren stieg die Inanspruchnahmequote in Westdeutschland wieder schrittweise an und erreichte 2024 das präpandemische Niveau, während sie in Ostdeutschland stagnierte. Im gesamten Zeitraum lag die Quote der Inanspruchnahme zahnärztlicher Kontrolluntersuchungen in Ostdeutschland deutlich höher als in Westdeutschland. Dieser Unterschied verringerte sich im Zeitverlauf von fast 10 Prozentpunkten im Jahr 2015 auf etwa 5 Prozentpunkte im Jahr 2024 (Abb. 3).

**Abb. 4:**
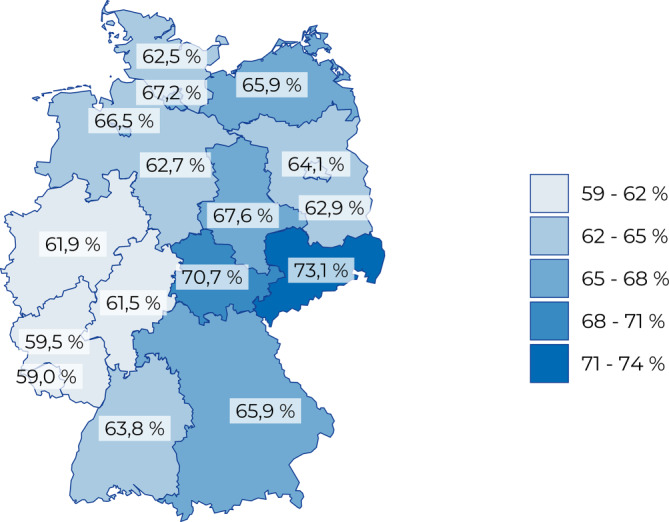
Quote der Inanspruchnahme zahnärztlicher Kontrolluntersuchungen (mindestens eine Abrechnung der BEMA 01, 151, 152, 153, 154 oder 155 im Kalenderjahr) nach Bundesland bei Erwachsenen ab 20 Jahren 2024; in Prozent (%). Quelle: vertragszahnärztliche Abrechnungsdaten der Kassenzahnärztlichen Bundesvereinigung (*KZBV*) und KM6-Statistik. (*BEMA* Einheitlicher Bewertungsmaßstab für zahnärztliche Leistungen)

Abb. 4 veranschaulicht die Quote der Inanspruchnahme zahnärztlicher Kontrolluntersuchungen nach Bundesland im Jahr 2024. Die Karte zeigt, dass die Inanspruchnahmequote in Sachsen, Thüringen, Sachsen-Anhalt, Hamburg, Bremen, Mecklenburg-Vorpommern, Bayern und Berlin über dem Bundesdurchschnitt von 64,0 % lag. In Baden-Württemberg, Brandenburg, Niedersachsen, Schleswig-Holstein, Nordrhein-Westfalen, Hessen, Rheinland-Pfalz sowie im Saarland lag die Inanspruchnahme unter dem Bundesdurchschnitt. Die höchste Inanspruchnahmequote verzeichnete Sachsen mit 73,1 %, die niedrigste das Saarland mit 59,0 %; dies entspricht einem Unterschied von fast 15 Prozentpunkten (Abb. 4).

### Sensitivitätsanalysen

Laut den Ergebnissen der Hauptanalyse (mindestens eine Abrechnung der BEMA 01, 151, 152, 153, 154 oder 155 im Kalenderjahr) betrug die Quote der Inanspruchnahme zahnärztlicher Kontrolluntersuchungen bei Erwachsenen ab 20 Jahren in 2024 64,0 % (Abb. 1 und Onlinematerial Tabelle A1). Wurde die Analyse auf die Abrechnung der BEMA 01 beschränkt (1. Sensitivitätsanalyse), fiel die Inanspruchnahmequote mit 63,4 % geringfügig niedriger aus. Wurde zusätzlich die BEMA Ä1 in die Auswertung einbezogen (2. Sensitivitätsanalyse), lag die Inanspruchnahmequote mit 68,1 % um 4 Prozentpunkte höher. Diese Größenverhältnisse waren für alle untersuchten Kalenderjahre zu beobachten (Onlinematerial Tabelle A1).

Wird der Blick auf die Gruppe der Hochaltrigen (ab 75 Jahre) gerichtet, lag die Quote der Inanspruchnahme zahnärztlicher Kontrolluntersuchungen in der Hauptanalyse (mindestens eine Abrechnung der BEMA 01, 151, 152, 153, 154 oder 155 im Kalenderjahr) im Jahr 2024 bei 63,8 % (Abb. 2 und Onlinematerial Abbildung A1). Wurde die Auswertung auf die BEMA 01 beschränkt (1. Sensitivitätsanalyse), lag die Inanspruchnahmequote bei 60,1 %; das heißt, dass die bei Hochaltrigen in Pflegeeinrichtungen oder im häuslichen Umfeld erbrachten Kontrolluntersuchungen im Jahr 2024 3,7 % an der Gesamtquote der 75-Jährigen und Älteren betrugen. Dieser Anteil war im Zeitverlauf mit etwa 3,0 % relativ stabil. Seit 2023 (3,5 %) zeichnete sich ein Aufwärtstrend ab; das bedeutet, dass die Quote der Inanspruchnahme zahnärztlicher Kontrolluntersuchungen in der Hauptanalyse etwas stärker angestiegen ist als in der Sensitivitätsanalyse (Onlinematerial Abbildung A1).

## Diskussion

Ziel dieser Arbeit war es, basierend auf den vertragszahnärztlichen Abrechnungsdaten der KZBV die zeitliche Entwicklung der Quote der Inanspruchnahme zahnärztlicher Kontrolluntersuchungen nach Geschlecht, Alter und Region (Ost/West) zu beleuchten. Ergänzend wurden Unterschiede in der Inanspruchnahmequote nach Bundesland für 2024 berichtet. Eine Literaturrecherche hat gezeigt, dass für Deutschland Analysen zur Inanspruchnahme zahnmedizinischer Leistungen auf Basis von GKV-Routinedaten existieren [11–14], aber nicht mit Fokus auf die Inanspruchnahme zahnärztlicher Kontrolluntersuchungen. Mit dem vorliegenden Beitrag wird somit eine bislang bestehende Forschungslücke adressiert.

### Zusammenfassung und Einordnung der Ergebnisse

Die Quote der Inanspruchnahme zahnärztlicher Kontrolluntersuchungen betrug in 2024 64,0 %. Dies entspricht einer Nichtinanspruchnahmequote von 36,0 %. Folglich hat mehr als jede dritte Person keine zahnärztliche Kontrolluntersuchung in Anspruch genommen. Im Untersuchungszeitraum verlief die Inanspruchnahmequote bis 2019 relativ konstant, sank im ersten COVID-19-Pandemiejahr 2020 und stieg in den Folgejahren auf das Ausgangsniveau in 2024 an. In diesem Zusammenhang sind Studien zu berücksichtigen, die zeigen, dass gesundheitliche Versorgungsleistungen während der COVID-19-Pandemie seltener in Anspruch genommen wurden [15]. Zahnärztliche und fachärztliche Kontrolltermine wurden am häufigsten abgesagt. Grund dafür war oft die Angst vor einer Ansteckung mit dem Erreger SARS-CoV‑2 [15].

Frauen nahmen zahnärztliche Kontrolluntersuchungen im gesamten Untersuchungszeitraum deutlich häufiger in Anspruch als Männer. Dass Frauen Vorsorge- und Früherkennungsuntersuchungen häufiger in Anspruch nehmen als Männer, zeigt sich z. B. auch bei der Teilnahme am gesetzlichen Gesundheits-Check-up [16] sowie am Darmkrebs-Screening-Programm [17]. Eine mögliche Erklärung hierfür ist das in anderen Studien beschriebene gesundheitsbewusstere Verhalten von Frauen [18, 19]. Bei der Inanspruchnahme von Zahnvorsorgeuntersuchungen dürfte außerdem eine Rolle spielen, dass Frauen höhere ästhetische Ansprüche in Bezug auf ihre Zähne haben, wie Studien aus Europa zeigen [20]. Unterschiede zeigen sich hier bereits im Kindes- und Jugendalter: Mädchen erhalten im Vergleich zu Jungen häufiger kieferorthopädische Behandlungen zur Korrektur von Fehlstellungen der Zähne [21, 22], was u. a. auf höhere ästhetische Ansprüche bei Mädchen zurückgeführt wird [22]. Auch bestimmte Persönlichkeitsmerkmale tragen zu einer höheren Inanspruchnahme zahnärztlicher Leistungen bei, z. B. Gewissenhaftigkeit [23]. Im Vergleich zu Männern sind Frauen in der Regel gewissenhafter und dadurch besser organisiert [24]. Dies dürfte mit erklären, warum Frauen Vorsorgetermine häufiger vereinbaren und wahrnehmen, nicht nur für sich selbst, sondern auch für ihre Kinder [25].

Ein weiteres Ergebnis dieser Arbeit ist, dass Personen im späten mittleren (45–64 Jahre) und höheren Erwachsenenalter (65–74 Jahre) im gesamten Untersuchungszeitraum die höchste Quote der Inanspruchnahme zahnärztlicher Kontrolluntersuchungen aufwiesen. Das ist plausibel, da gesundheitliche Themen mit zunehmendem Alter an Bedeutung gewinnen [26]. Junge Erwachsene (20–34 Jahre) wiesen in allen Kalenderjahren die geringste Inanspruchnahmequote auf. Für diese altersbezogenen Unterschiede kommen verschiedene Erklärungsansätze in Betracht. Aus der Entwicklungspsychologie ist bekannt, dass das junge Erwachsenenalter mit diversen Veränderungen und Herausforderungen einhergeht [26]. So müssen junge Erwachsene bspw. lernen, sich um die eigene Gesundheit zu kümmern. In die Phase des jungen Erwachsenenalters fällt in der Regel auch die Geburt des ersten Kindes [27]. Eine Schwangerschaft geht mit zahlreichen körperlichen Veränderungen einher [28]. Auch die Mundgesundheit wird durch die hormonelle Umstellung beeinflusst, wodurch das Risiko für Frühgeburten und das Auftreten oraler Erkrankungen, darunter Gingivitis, Parodontitis und Karies, steigen [29]. Ein Teil der Schwangeren nimmt Zahnvorsorgeuntersuchungen aber nicht in Anspruch, wie eine dänische Studie zeigt [30]. Mütter und Väter sind Vorbilder für ihre Kinder und prägen ihr Verhalten bis ins Erwachsenenalter hinein [26]. Das gilt auch für das Mundgesundheitsverhalten mit z. B. regelmäßigen Kontrolluntersuchungen in der zahnärztlichen Praxis [31, 32]. Vor diesem Hintergrund erscheinen gezielte Maßnahmen sinnvoll, um die Inanspruchnahme zahnärztlicher Kontrolluntersuchungen insbesondere im jungen (20–34 Jahre) sowie frühen mittleren Erwachsenenalter (35–44 Jahre) zu erhöhen.

Darüber hinaus wiesen Hochaltrige (ab 75 Jahren) eine im Vergleich zur Gesamtquote geringere Inanspruchnahmequote auf. Der Rückgang in der Inanspruchnahmequote im ersten COVID-19-Pandemiejahr 2020 war bei ihnen mit rund 4 Prozentpunkten am stärksten ausgeprägt. Eine mögliche Erklärung hierfür könnte sein, dass ältere Menschen aufgrund bestehender Vorerkrankungen und eines schlechteren allgemeinen Gesundheitszustands seltener zahnärztliche und ärztliche Leistungen in Anspruch genommen haben [15, 33]. Anschließend stieg die Inanspruchnahmequote bei den Hochaltrigen wieder allmählich an und erreichte in 2024 fast das Niveau der Gesamtquote mit 64,0 %. Bei der Mehrheit der Hochaltrigen fanden Kontrolluntersuchungen in der zahnärztlichen Praxis statt. Die in Pflegeeinrichtungen oder im häuslichen Umfeld durchgeführten Kontrolluntersuchungen machten von 2015 bis 2022 etwa 3 % der Gesamtquote der 75-Jährigen und Älteren aus, während sich in den Jahren danach ein Aufwärtstrend abzeichnete (2023: 3,5 %; 2024: 3,7 %). In diesem Zusammenhang zu berücksichtigen ist, dass die Mundgesundheit von Pflegebedürftigen seit einigen Jahren im Fokus der KZBV steht [34]. So wurden in 2018 5 zusätzliche Gebührenziffern zur Verhütung von Munderkrankungen bei Pflegebedürftigen und Menschen mit Behinderungen in den BEMA aufgenommen [35]. Ziel war, die Mundgesundheit dieser vulnerablen Gruppe zu verbessern. Pflegende Angehörige und Pflegekräfte können seitdem besser in die Mundgesundheitsaufklärung und in den individuellen Mundgesundheitsplan einbezogen werden [34]. Dies könnte sich indirekt auch positiv auf die Inanspruchnahme zahnärztlicher Kontrolluntersuchungen ausgewirkt haben. Dennoch machten aufsuchende Kontrolluntersuchungen bei Hochaltrigen nur einen kleinen Anteil an ihrer Gesamtquote aus. Dies weist auf fortbestehende Zugangsbarrieren hin, etwa eingeschränkte Mobilität, Pflegebedürftigkeit und institutionelle Hürden. Zugleich unterstreicht es die Notwendigkeit, präventive Versorgungspfade stärker in Pflegeeinrichtungen und im häuslichen Setting zu verankern sowie strukturelle Barrieren, etwa in Kooperationsstrukturen, Angebotsdichte, barrierearmer Organisation, weiter abzubauen. Künftige Forschungsarbeiten müssen zeigen, ob sich dieser aufgezeigte positive Trend in den nächsten Jahren fortsetzt.

Regional zeigten sich deutliche Unterschiede: Im gesamten Untersuchungszeitraum lag die Inanspruchnahmequote zahnärztlicher Kontrolluntersuchungen in Ostdeutschland über derjenigen in Westdeutschland. Besonders deutlich war im Jahr 2024 der Unterschied zwischen Sachsen mit der höchsten und dem Saarland mit der niedrigsten Inanspruchnahmequote; er betrug fast 15 Prozentpunkte. Ost-West-Unterschiede in der Mundgesundheit und im Mundgesundheitsverhalten sind seit Langem bekannt. Eine mögliche Erklärung wird auf die staatlich organisierte Gesundheitsfürsorge in der DDR zurückgeführt, die mit einer besseren Mundgesundheit der Bevölkerung einherging [36]. Der BARMER Zahnreport 2022, der die Entwicklung der vertragszahnärztlichen Versorgung zum Schwerpunkt hatte, konnte in dieser Hinsicht zeigen, dass sich die Ost-West-Unterschiede bei Personen verlieren, die nach der Wiedervereinigung aufgewachsen sind [37]. Die in dieser Arbeit präsentierten Ergebnisse stehen damit im Einklang und zeigen, dass sich die Unterschiede in der Inanspruchnahme zahnärztlicher Kontrolluntersuchungen zwischen den Landesteilen aufgrund eines Rückgangs der Quote in Ostdeutschland verringern. Ein weiterer Grund hierfür könnte sein, dass sich die Angebotsdichte an Zahnärzt:innen in den beiden Landesteilen in dem hier betrachteten Zeitraum von 2015 bis 2024 unterschiedlich entwickelt hat: Während die Zahl der Zahnärzt:innen in Ostdeutschland rückläufig ist, hat sie in Westdeutschland sukzessive zugenommen [38].

### Vergleich mit Prävalenzen zu zahnärztlichen Kontrolluntersuchungen in Befragungsdaten

Daten zur Inanspruchnahme zahnärztlicher Kontrolluntersuchungen werden in der Deutschen Mundgesundheitsstudie (DMS) des Instituts der Deutschen Zahnärzte (IDZ) und in den Gesundheitssurveys des Robert Koch-Instituts (RKI) erhoben [33, 39, 40]. Mit Blick auf den hier betrachteten Untersuchungszeitraum von 2015 bis 2024 liegen Vergleichswerte für die Jahre 2022 und 2023 aus der bundesweiten Studie Gesundheit in Deutschland aktuell (GEDA) des RKI vor [33]. Laut den Ergebnissen haben in 2022 66,5 % und in 2023 68,0 % der Erwachsenen ab 18 Jahren zahnärztliche Kontrolluntersuchungen im Jahr vor der Befragung in Anspruch genommen. Damit liegen die Prävalenzen aus der GEDA-Studie um rund 4 Prozentpunkte höher im Vergleich zu den Quoten, die auf den vertragszahnärztlichen Abrechnungsdaten der KZBV basieren und Erwachsene ab 20 Jahren einschließen (2022: 62,8 %; 2023: 64,0 %). Übereinstimmend zeigten sich in der GEDA-Studie Unterschiede nach Geschlecht und Alter zugunsten von Frauen bzw. Personen im späten mittleren (45–64 Jahre) und höheren Erwachsenenalter (65–74 Jahre). Junge Erwachsene (20–34 Jahre) und Hochaltrige (ab 75 Jahre) wiesen die geringste Inanspruchnahmequote auf [33]. Ein Vorteil von Surveydaten ist, dass neben Alter und Geschlecht auch sozioökonomische Merkmale der Teilnehmenden, wie Bildungsabschluss und Einkommen, erhoben werden. So konnte auf Basis der GEDA-Studie gezeigt werden, dass ausgeprägte sozioökonomische Unterschiede in der Inanspruchnahme zahnärztlicher Kontrolluntersuchungen bestehen [33, 39]. Studien sprechen dafür, dass sozioökonomisch besser gestellte Personen stärker durch Präventionsangebote des Gesundheitssystems, wozu auch das Bonusheft für Zahnvorsorgeuntersuchungen gehört, angesprochen werden [41]. Dadurch besteht das Risiko, dass gesundheitliche Ungleichheiten in der Mundgesundheit und im Mundgesundheitsverhalten durch derartige Angebote noch verstärkt werden [42].

### Stärken und Limitationen

Bei der Datengrundlage der vorliegenden Arbeit sind sowohl Stärken als auch Limitationen zu berücksichtigen, die im Vergleich zu den zur Einordnung herangezogenen Befragungsdaten der GEDA-Studie dargestellt werden: Die Analysen basieren auf Routinedaten, die primär für die Abrechnung zahnärztlicher Leistungen bei gesetzlich Versicherten codiert wurden [10]. Für die Fragestellung dieser Arbeit wurden sie als Sekundärdaten pro Kalenderjahr analysiert. Ein genereller Vorteil von Abrechnungsdaten ist, dass sie in der Regel auf großen Fallzahlen basieren, tiefergehende räumliche Auflösung ermöglichen und zumeist häufiger und schneller zur Verfügung stehen [43]. Von Nachteil für wissenschaftliche Auswertungen ist dagegen, dass sie Leistungsansprüche abbilden und dass u. a. finanzielle Anreize dazu führen können, dass sich die abgerechneten Daten in einem gewissen Umfang von der Versorgungswirklichkeit unterscheiden [10]. So kann z. B. nicht gänzlich ausgeschlossen werden, dass gelegentlich auch bei Abrechnung der Position Ä1 eine eingehende Untersuchung stattgefunden hat, die allerdings nicht als BEMA 01 angesetzt werden kann, weil die letzte Abrechnung noch keine 4 Monate zurückliegt. Andererseits ist der im Rahmen der BEMA 01 tatsächlich durchgeführte Leistungsumfang unklar. Darüber hinaus liegen nur begrenzt Informationen zu sozioökonomischen Merkmalen, wie Bildungsabschluss oder Einkommen vor, sodass diese häufig nur indirekt, beispielsweise über den German Index of Socioeconomic Deprivation (GISD; [44]), auf räumlicher Ebene abgebildet werden können. Die analysierten KZBV-Daten basieren auf Routinedaten der vertragszahnärztlichen Versorgung und umfassen die Gesamtheit der gesetzlich Versicherten ab 20 Jahren. Sie stellen damit keine Stichprobe, sondern eine nahezu vollständige Erfassung der GKV-Versicherten dar (ca. 87–90 % der Bevölkerung). Da sich Größe und Zusammensetzung der Versichertenpopulation im Zeitverlauf verändern [43], bilden die Ergebnisse jährliche Querschnitte einer dynamischen Grundgesamtheit ab. Erfasst werden ausschließlich Leistungen der vertragszahnärztlichen Versorgung innerhalb der GKV. Privat versicherte und beihilfeberechtigte Personen sowie privat finanzierte Leistungen außerhalb des GKV-Leistungskatalogs sind nicht enthalten. Auch Gruppen mit teilweise außerhalb des vertragszahnärztlichen Systems organisierter Versorgung (z. B. Justizvollzug oder militärische Versorgung) können unterrepräsentiert sein. Die Ergebnisse sind daher nicht repräsentativ für die Gesamtbevölkerung in Deutschland.

Auch Befragungsdaten können zur Untersuchung des Inanspruchnahmeverhaltens herangezogen werden, wie in der hier zitierten GEDA-Studie. Surveydaten basieren auf Selbstauskünften und sind dadurch anfällig für gewisse Verzerrungen, z. B. durch selektive Nichtteilnahme (Nonresponse-Bias; [45]), Erinnerungseffekte (Recall-Bias; [46]) oder soziale Erwünschtheit im Antwortverhalten [47]. So könnte der Anteil der Personen, die nach eigenen Angaben in den letzten 12 Monaten eine zahnärztliche Kontrolluntersuchung wahrgenommen haben, in der telefonischen GEDA-Befragung aufgrund von sozial erwünschtem Antwortverhalten tendenziell überschätzt sein [47]. Für Personen mit gesundheitlichen Einschränkungen ist die Teilnahme an Surveys, die sich an die Allgemeinbevölkerung richten, manchmal nur schwer oder gar nicht möglich [48]. Andererseits schließt die GEDA-Studie Personen unabhängig von ihrem Krankenversicherungsstatus ein. Da privat Versicherte in der Regel über eine höhere Bildung verfügen [43], das wiederum mit einem höheren Inanspruchnahmeverhalten assoziiert ist, sind höhere Quoten als bei gesetzlich Versicherten zu erwarten [33, 39]. Durch die Anwendung eines studienspezifischen Gewichtungsfaktors bei GEDA-Analysen wird zudem ein hohes Maß an Repräsentativität für die in Deutschland lebende erwachsene Wohnbevölkerung erreicht [49]. Für die niedrigeren Inanspruchnahmequoten bei den in dieser Arbeit analysierten KZBV-Daten im Vergleich zu GEDA sind vor diesem Hintergrund verschiedene Erklärungsansätze denkbar. Beide Datenquellen liefern komplementäre Informationen, die sich den tatsächlichen Quoten mit ihren jeweiligen Stärken und Schwächen auf verschiedenen Wegen annähern.

Die vorliegende Studie beschränkt sich auf deskriptive Auswertungen. Weiterführende inferenzstatistische Analysen (z. B. Poisson-Regression) könnten zusätzliche Erkenntnisse liefern. Zudem könnten weitere Stratifizierungen interessant sein, etwa zur Untersuchung von Unterschieden zwischen städtischen und ländlichen Regionen oder nach regionaler sozioökonomischer Deprivation [44].

## Fazit und Ausblick

Regelmäßige zahnärztliche Kontrolluntersuchungen wirken sich positiv auf die Mundgesundheit aus und verringern das Risiko, beispielsweise an Karies und Parodontitis zu erkranken [2]. Für Deutschland lagen auf der Basis von Routinedaten bislang keine Analysen zum zeitlichen Verlauf der Inanspruchnahme zahnärztlicher Kontrolluntersuchungen bei Erwachsenen vor. Dieser Beitrag schließt damit eine Forschungslücke. Laut den Ergebnissen war die Inanspruchnahmequote zwischen 2015 und 2019 mit rund 64,0 % relativ stabil, sank im ersten COVID-19-Pandemiejahr 2020 um rund 2 Prozentpunkte ab und stieg bis 2024 wieder schrittweise auf das präpandemische Niveau an. Vor dem Hintergrund, dass in 2024 mehr als jede dritte erwachsene Person keine zahnärztliche Kontrolluntersuchung in Anspruch genommen hat, ist die zahnärztliche Versorgung in Deutschland nach wie vor stark auf Therapie ausgerichtet [8]. Die Mundgesundheitsziele für Deutschland 2030, veröffentlicht von der Bundeszahnärztekammer (BZÄK), sehen vor, die Prävention zu stärken [50]. Hierzu gehört auch, die Quote der Inanspruchnahme zahnärztlicher Kontrolluntersuchungen in der Bevölkerung zu erhöhen [50]. Der vorliegende Beitrag weist wichtige Risikogruppen im Erwachsenenalter aus, etwa Männer, Personen im jungen und frühen mittleren Lebensalter, Hochaltrige sowie Personen in Westdeutschland, insbesondere in den Bundesländern Nordrhein-Westfalen, Hessen, Rheinland-Pfalz und im Saarland.

## Supplementary Information

Onlinematerial: Ergebnisse Sensitivitätsanalysen

## Data Availability

Für die Studie wurden anonymisierte Abrechnungsdaten der Kassenzahnärztlichen Bundesvereinigung (KZBV) verwendet. Dieser Datensatz kann nicht öffentlich zugänglich gemacht werden. Anfragen zum aggregierten Datensatz, der den Ergebnissen zugrunde liegt, können per E‑Mail an die korrespondierende Autorin gestellt werden.
