# Supplementary material for: Utilisation of dental check-ups in Germany—prevalence and trends based on routine statutory health insurance data
Source: Bundesgesundheitsblatt Gesundheitsforschung Gesundheitsschutz. 2026 Jun 9;69(7):803–12. [Article in German] doi: 10.1007/s00103-026-04253-3 (PMC13323270; doi:10.1007/s00103-026-04253-3)
Supplement: Supplementary file 1 — Onlinematerial: Ergebnisse Sensitivitätsanalysen [file 103_2026_4253_MOESM1_ESM.pdf]

Onlinematerial

Tabelle A1 Zeitlicher Verlauf der Inanspruchnahmequote nach Gebührennummern des Einheitlichen Bewertungsmaßstabs für zahnärztliche Leistungen (BEMA) bei Erwachsenen ab 20 Jahren 2015 bis 2024; in Prozent (%). Quelle: Vertragszahnärztliche Abrechnungsdaten der Kassenzahnärztlichen Bundesvereinigung (KZBV)

	2015	2016	2017	2018	2019	2020	2021	2022	2023	2024
	%	%	%	%	%	%	%	%	%	%
**Hauptanalyse [1]**	64,8	64,7	64,4	64,0	64,4	62,1	63,3	62,8	63,7	64,0
**1. Sensitivitätsanalyse [2]**	64,3	64,1	63,9	63,5	63,8	61,6	62,7	62,3	63,1	63,4
**2. Sensitivitätsanalyse [3]**	69,8	69,6	69,3	68,8	69,0	66,6	67,5	67,2	67,9	68,1

[1] mindestens eine Abrechnung der BEMA 01, 151, 152, 153, 154 oder 155 im Kalenderjahr

[2] mindestens eine Abrechnung der BEMA 01 im Kalenderjahr

[3] mindestens eine Abrechnung der BEMA 01, 151, 152, 153, 154, 155 oder Ä1 im Kalenderjahr

Abbildung A1 Zeitlicher Verlauf der Inanspruchnahmequote zahnärztlicher Kontrolluntersuchungen nach Gebührennummern des Einheitlichen Bewertungsmaßstabs für zahnärztliche Leistungen (BEMA) bei Hochaltrigen ab 75 Jahren 2015 bis 2024; in Prozent (%). Quelle: Vertragszahnärztliche Abrechnungsdaten der Kassenzahnärztlichen Bundesvereinigung (KZBV)

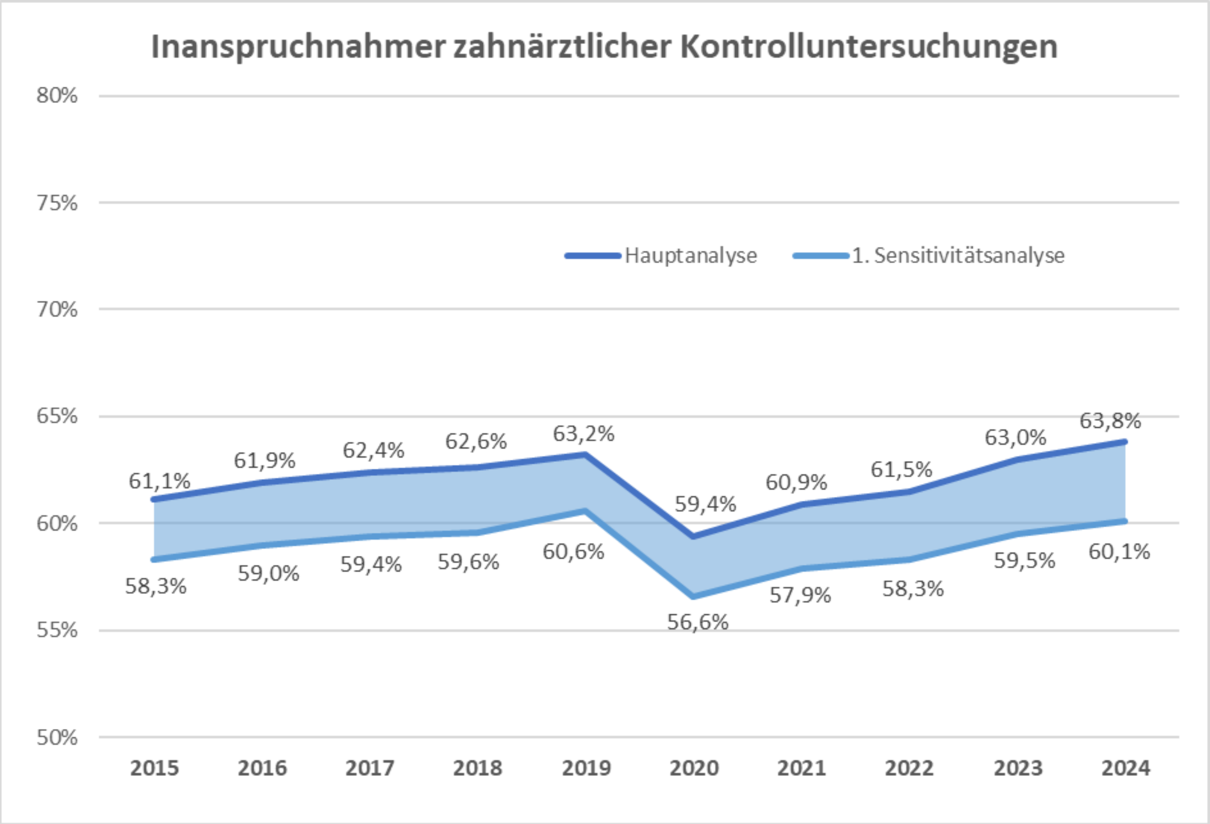

Hauptanalyse: mindestens eine Abrechnung der BEMA 01, 151, 152, 153, 154 oder 155 im Kalenderjahr

1. Sensitivitätsanalyse: mindestens eine Abrechnung der BEMA 01 im Kalenderjahr
